# The *OPRM1* A118G polymorphism modulates the descending pain modulatory system for individual pain experience in young women with primary dysmenorrhea

**DOI:** 10.1038/srep39906

**Published:** 2017-01-06

**Authors:** Shyh-Yuh Wei, Li-Fen Chen, Ming-Wei Lin, Wei-Chi Li, Intan Low, Ching-Ju Yang, Hsiang-Tai Chao, Jen-Chuen Hsieh

**Affiliations:** 1 Institute of Brain Science, School of Medicine, National Yang-Ming University, Taipei, Taiwan; 2 Integrated Brain Research Unit, Department of Medical Research, Taipei Veterans General Hospital, Taipei, Taiwan; 3 Institute of Biomedical Informatics, School of Medicine, National Yang-Ming University, Taipei, Taiwan; 4 Institute of Public Health, School of Medicine, National Yang-Ming University, Taipei, Taiwan; 5 Department of Obstetrics and Gynecology, School of Medicine, National Yang-Ming University, Taipei, Taiwan; 6 Department of Obstetrics and Gynecology, Taipei Veterans General Hospital, Taipei, Taiwan

## Abstract

The mu-opioid receptor (*OPRM1*) A118G polymorphism underpins different pain sensitivity and opioid-analgesic outcome with unclear effect on the descending pain modulatory system (DPMS). Primary dysmenorrhea (PDM), the most prevalent gynecological problem with clear painful and pain free conditions, serves as a good clinical model of spontaneous pain. The objective of this imaging genetics study was therefore to explore if differences in functional connectivity (FC) of the DPMS between the *OPRM1* A118G polymorphisms could provide a possible explanation for the differences in pain experience. Sixty-one subjects with PDM and 65 controls participated in the current study of resting-state functional magnetic resonance imaging (fMRI) during the menstruation and peri-ovulatory phases; blood samples were taken for genotyping. We studied 3 aspects of pain experience, namely, mnemonic pain (recalled overall menstrual pain), present pain (spontaneous menstrual pain), and experimental pain (thermal pain) intensities. We report that G allele carriers, in comparison to AA homozygotes, exhibited functional hypo-connectivity between the anterior cingulate cortex (ACC) and periaqueductal gray (PAG). Furthermore, G allele carriers lost the correlation with spontaneous pain experience and exhibited dysfunctional DPMS by means of PAG-seeded FC dynamics. This *OPRM1* A118G-DPMS interaction is one plausible neurological mechanism underlying the individual differences in pain experience.

The experience of pain and the clinical response to opioid analgesics varies among people. The analgesic effects of opioids are substantially mediated by the mu-opioid receptor (OPRM1) in the central nervous system^1^. A single nucleotide polymorphism producing an adenine (A)-to-guanine (G) substitution at codon118 (A118G) in the human *OPRM1* gene is associated with reduced expression of the *OPRM1* gene^2^, hypersensitivity to pain^3^, and an increased consumption of analgesics for clinical pain^4^.

The periaqueductal gray (PAG), enriched with opioidergic neurons^1^, acts as a critical hub in the neuraxis of the descending pain modulatory system (DPMS)^5^ that is pivotal for pain relief^6^. Nevertheless, to the best of our knowledge, no study has addressed how the *OPRM1* A118G polymorphism modulates the DPMS to relate the individual pain experience. In the current imaging genetics study (using neuroimaging as an endophenotypic assay to evaluate genetic associations, with pain as an environmental stress) we investigated the neural network mechanisms of the *OPRM1* A118G polymorphism for the central modulation of pain in otherwise healthy women with primary dysmenorrhea (PDM).

PDM, defined as menstrual pain without discernable organic causes, is the most prevalent gynecological problem with clear painful (menstrual phase) and pain free (e.g., peri-ovulatory phase) conditions and serves as a good clinical model of spontaneous pain^7^. PDM is considered as a genuine chronic pain condition^8^ because the neural substrates of pain matrix may be altered in long-term PDM in the context of glucose metabolism, regional homogeneity and activity, *state-* and *trait-related* structural changes^9^,^10^,^11^,^12^,^13^. *State-related* changes are menstrual pain-primed, whereas *trait-related* changes exist even without symptoms. Alterations of regional gray matter volume in PDM substantially involved the DPMS (PAG, insula, ACC/medial prefrontal cortex [mPFC], and etc.)^10^. It is suggested that the maladaptive functional connectivity (FC) in the DPMS (PAG-ACC/mPFC and PAG-supplementary motor area [SMA]) in young PDM subjects may underpin the central susceptibility to subsequent development of various chronic pain disorders later in life^7^. Such PAG-ACC/mPFC hypo-FC is a co-terminal to many functional pain disorders, while altered SMA FC is common in chronic pelvic pain disorders^7^,^14^. Notably, there may even be developmental attributions for PDM owing to its association with a high prevalence of normal variants^15^.

Using PAG seed-based FC approach, studies have revealed abnormal DPMS neurodynamics in patients with chronic low back pain^16^, chronic myofascial pain^17^, fibromyalgia^18^, migraine^19^, and PDM^7^; the alterations of PAG-seeded FC dynamics relate patients’ pain experience. We further reported that the PAG-seeded DPMS FC patterns in pain-free controls are altered in women with PDM in a genotype-specific manner^20^. We set out in the current study to investigate the effect of *OPRM1* A118G polymorphism on the PAG-seeded DPMS FC dynamics throughout the menstrual cycle and correlate different aspects of pain experience with the PAG-seeded FCs in the PDM subjects. Here, we studied 3 aspects of pain experience, namely, mnemonic pain (recalled overall menstrual pain), present pain (spontaneous menstrual pain), and experimental pain (thermal pain) intensities. We hypothesized that the *OPRM1* A118G polymorphism may be associated with disparate PAG-seeded FCs (networks) in the DPMS that may explicate the individual differences in pain experience.

## Results

### Demographic data and baseline information

Sixty-one PDM subjects (AA *n* = 26, AG *n* = 31, GG *n* = 4; 23.2 ± 2.27 years of age) and 65 age- and education-matched healthy female controls (AA *n* = 23, AG *n* = 36, GG *n* = 6; 23.8 ± 2.36 years of age) of the same Chinese ethnicity were eligible for imaging genetics studies. No significant differences between the PDM subjects and the controls were detected for the *OPRM1* A118G polymorphism (*p* = 0.660) and the A118G-genotype distribution did not deviate from the Hardy–Weinberg equilibrium in both PDM (*p* = 0.19) and control group (*p* = 0.13). AG heterozygotes and GG homozygotes were considered as a single genotype (“G allele carriers”) based on their similar clinical characteristics^4^. There were no significant group or genotype differences with regard to age, years of menstruation, average days of 1 menstrual cycle, or Edinburgh Handedness Inventory scores (Table 1).

All 61 PDM subjects in this neuroimaging study had a long history of menstrual pain (mean ± SD = 8.95 ± 2.82 years), with the pain lasting approximately 1 to 3 days during one menstrual cycle (mean ± SD = 1.93 ± 0.79 days). Thirty-four PDM subjects (55.7%) reported absences from school or work as a result of debilitating menstrual pain, and 28 PDM subjects (45.9%) used over-the-counter analgesics on occasion. There was no significant genotype difference with regard to history of menstrual pain (*p* = 0.819), duration of menstrual pain (*p* = 0.931), absences from school or work (*p* = 0.432), or analgesics taken (*p* = 0.813).

### Psychological measurements

The mnemonic experience (mean ± SD = 34.84 ± 13.92) and the present experience (mean ± SD = 31.66 ± 13.02) of menstrual pain, as assessed by scores on the McGill Pain Questionnaire, confirmed that the PDM subjects experienced moderate to severe menstrual pain. The pain rating index (PRI) of present pain was significantly lower than that of mnemonic pain (*p* = 0.039; mnemonic pain is usually rated higher than present pain^7^,^21^). Notably, G allele carriers rated present pain sub-significantly higher than AA homozygotes under similar mnemonic pain experience (Table 1).

### Serum gonadal hormone measurements

There were phase differences for all 3 gonadal hormones. No group or *OPRM1* genotype difference was noted (Table 2). As gonadal hormones may affect the resting-state FC^22^,^23^,^24^, the hormone fluctuations were regressed out as covariates of non-interest during the subsequent image processing.

### Thermal quantitative sensory test

The PDM subjects had lower heat-pain thresholds in the C7 (remote control area; significant) and T11 dermatomes (referral area of menstrual pain; sub-significant), suggesting central sensitization throughout the menstrual cycle as previously reported in white populations^25^,^26^,^27^. No significant genotype or menstrual cycle difference was noted (Table 3).

### Neuroimaging studies

#### PAG-seeded FC maps for the between-genotype and between-group differences

**Figure 1:**
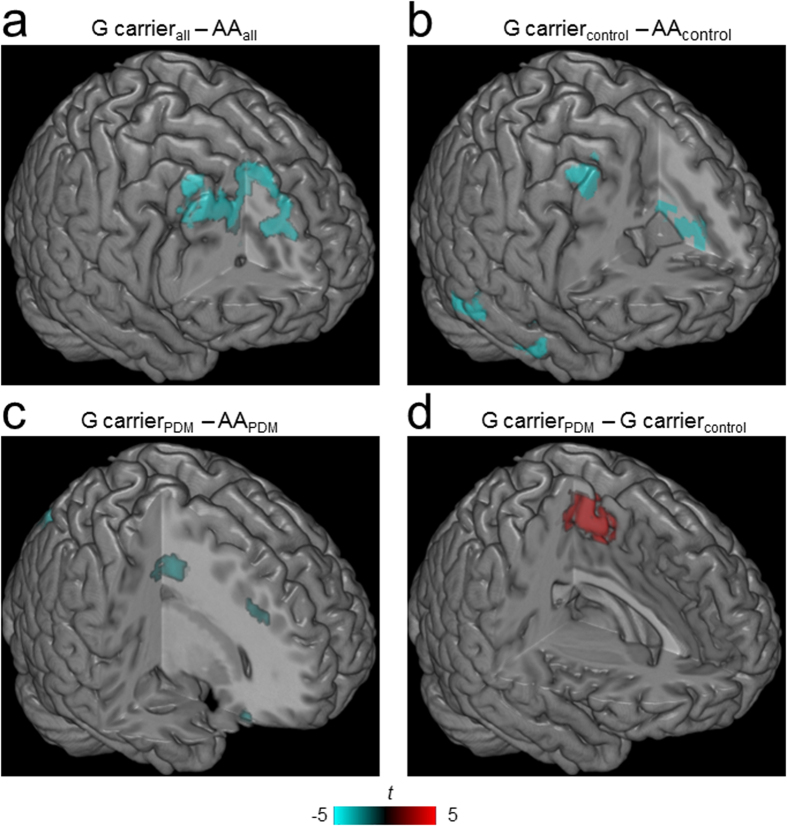
The *OPRM1* A118G polymorphism is associated with diverse functional expressions of the pain modulatory system during menstruation in primary dysmenorrheic women and controls. Regions showed significant FC with the PAG of between-genotype (**a–c**) and between-group (**d**) comparisons. Each region’s coordinates are listed in Table 4. (**a**) Overall, G allele carriers exhibited decreased PAG-ACC/mPFC and -dlPFC FC compared with AA homozygotes. (**b**) In healthy subjects, G allele carriers exhibited decreased PAG-ACC, -dlPFC and -middle temporal gyrus FC compared with AA homozygotes. (**c**) In PDM subjects, G allele carriers exhibited decreased PAG-ACC/mPFC, -orbitofrontal cortex and -superior parietal lobule FC compared with AA homozygotes. (**d**) Only among G allele carriers did PDM subjects exhibit increased PAG-supplementary motor area FC compared with the controls. Results in (**a**,**b**,**d**) were thresholded at the uncorrected voxel level *p* = 0.005, followed by the FWE-corrected cluster level *p* = 0.05; results in **c** were thresholded at the uncorrected voxel level *p* = 0.005, voxels >60. The color bar denotes the *t*-scores. Figures are displayed according to neurological convention (left = left). FC, functional connectivity; PAG, periaqueductal gray; ACC, anterior cingulate cortex; dlPFC, dorsolateral prefrontal cortex; mPFC, medial prefrontal cortex; PDM, primary dysmenorrhea.

In the overall between-genotype FC comparisons, G allele carriers showed decreased PAG-ACC/mPFC and PAG-dorsolateral prefrontal cortex (dlPFC) FCs compared with AA homozygotes. Among the control group, G allele carriers showed decreased PAG-ACC, PAG-dlPFC and PAG-temporal lobe FCs compared with AA homozygotes. Among the PDM subjects, G allele carriers showed decreased PAG-ACC/mPFC, -orbitofrontal cortex and -superior parietal lobule FCs compared with AA homozygotes at a lower threshold (uncorrected *p* = 0.005, voxels >60). Only among the G allele carriers, PDM subjects showed increased PAG-SMA FC compared with the controls (Fig. 1, Table 4).

#### Correlation analysis between functional connectivity and the pain rating index of the McGill Pain Questionnaire

During the menstruation, the AA homozygous PDM subjects exhibited positive correlations between their present experience of menstrual pain and PAG-seeded FC in the ACC, insula, secondary somatosensory cortex (S2), subthalamic nucleus, and cerebellum (Table 5). However, the PDM subjects with G allele showed no correlation between PAG-seeded FC and their experience of menstrual pain, either present or mnemonic.

#### Correlation analysis between functional connectivity and the heat-pain threshold of the quantitative sensory test

During both phases, only the PDM subjects with G allele exhibited negative correlations between PAG-cuneus FC and their heat-pain thresholds over both C7-dermatome and T11-dermatome (Table 6).

## Discussion

Individual differences in pain experience may stem from genotype-laden different pain processing and neuromodulatory mechanisms in the brain, especially the DPMS. Here we report that AA homozygotes, in comparison to G allele carriers, exhibited increased PAG-ACC FC that correlated with their spontaneous menstrual pain experience. The AA homozygous PDM demonstrated an active cortical modulation, whilst the PDM subjects with G allele showed dys-regulated DPMS. This *OPRM1* A118G-DPMS interaction can be one plausible neurological mechanism underlying the individual differences in pain experience.

In the overall between-genotype FC comparisons, G allele carriers (*vs*. AA homozygotes) showed hypo-FCs between the ACC, mPFC and dlPFC with PAG during menstruation (Fig. 1a). All of these FCs participate in the cortical modulation of pain^6^,^28^. The findings indicate an innately decreased endogenous modulation in G allele carriers even among the healthy subjects (Fig. 1b). This view is corroborated by the reported observation that G allele carriers require more self-administered analgesics after surgery^4^,^29^.

Using a relatively liberal statistics (uncorrected *p* = 0.005, voxels >60; compromising with relatively small sample size of AA homozygous PDMs *n* = 26 *vs.* G allele carrier PDMs *n* = 35) to unravel subtle genetic effect yet important information from the subset analyses, we observed significant differences in the between-genotype comparison among PDM subjects (Fig. 1c). The PAG-ACC, -mPFC, -orbitofrontal cortex, and -superior parietal lobule FCs are all known to substantially participate in the cortical modulation of pain^28^. The findings from the subset analyses of PDM subjects were corroborative to those from between-genotype comparisons of the controls (Fig. 1b) and overall subjects (Fig. 1a); all these between-genotype comparisons (overall, controls, and PDMs, respectively) showed consistent PAG-ACC hypo-FC in G allele carriers (Table 4). The ACC, enriched with opioidergic neurons^30^, is pivotal for the DPMS with respect to placebo, opioid analgesia^31^,^32^, and distraction from pain^33^,^34^. Therefore, the PAG-ACC FC is likely to be one plausible neurological mechanism underlying the *OPRM1* A118G polymorphism-associated genetic differences in pain experience. Our reasoning is supported by the correlations between present pain experience and the PAG-ACC FC only in the AA homozygous PDM subjects during menstruation, but not in the PDM subjects with G allele (Table 5). Collectively, our data suggest an active cortical modulation for present menstrual pain and may explain why AA homozygotes rated their present pain experience sub-significantly lower than G allele carriers.

In the between-group comparisons for each genotype, only the G allele carriers with PDM exhibited increased PAG-SMA FC during menstruation (Fig. 1d). Increased SMA activation has been noted in G allele carriers compared with AA homozygotes during painful electrical stimulation^35^. Such PAG-SMA FC is a co-terminal to many chronic pelvic pain disorders and may implicate a dysfunctional DPMS involving the medial motor cortex and PAG^7^,^14^. The findings suggest that G allele carries can be associated with higher vulnerability for the development of chronic pain. Furthermore, the negative correlations between the PAG-cuneus FC and experimental heat-pain thresholds over both C7- and T11-dermatomes throughout their menstrual cycle (*trait* correlation; Table 6) imply that the PAG-cuneus FC could predict the pain sensitivity. It has been suggested that the cuneus may have a connection to the PAG because stimulation of the area in rats leads to descending pain alleviation^36^, and the PAG-cuneus FC is engaged for pain modulation in PDM subjects^7^. However, the negative correlations in G allele carriers implicate maladaptive neuroplasticity. It is noteworthy that G allele carriers exhibited no correlation between their PRI and PAG-seeded FC. Collectively, the absence of *state* correlations and the presence of stationary *trait* correlations in G allele carriers may implicate subclinical dys-regulated pain modulation.

Unlike some previous genetic studies on the *OPRM1* A118G polymorphism, we did not observe differences in the heat-pain threshold. There are several explanations for these inconsistencies in the existing literature, including differences in sex proportion (sex by genotype interaction emerges for heat pain^37^), pain modalities (neither electrical^3^ nor pressure^37^ pain stimulation), and ethnicity (Asians are different from Caucasians in the G allele variability^38^ and in the phenotype of the *OPRM1* A118G polymorphism^39^). As the penetrance of genes can be greater at the level of brain biology than at the level of behavior^40^, neuroimaging bridges the mechanistic gap between gene and behavior^41^. Neuroimaging may be more sensitive than behavioral measurements, i.e., the neuroimaging findings can be sub-clinical without conspicuous behavioral manifestation^42^; therefore, the PDM subjects with G allele may show altered FC, but without conspicuous behavioral differences in their heat-pain thresholds.

Our study depicts that the *OPRM1* A118G polymorphism influences the DPMS FC dynamics during painful menstruation phase (*state*-related change) in the PDM subjects, but not during the pain-free peri-ovulatory phase (*trait*-related change), positing menstrual pain as a potential attributor of epigenetic regulation (Fig. 1 and Table 4). It has been reported that epigenetic modifications (DNA methylation in the frontal cortex) mostly happen during the period of adolescent and become stable in adults^43^. It is tempting to speculate that early PDM experiences might invoke epigenetic regulation of *OPRM1* A118G polymorphism, affect the frontal DPMS with long-term sensitization, and incur maladaptive neuroplasticity that eventually might lead to various functional pain disorders later in life in the vulnerable female subjects^7^. It should be born in mind that vulnerability to an illness, e.g., PDM, may stem not only from a single single-nucleotide polymorphism effect but also the interactions and contributions of multiple genes^44^. In line with this, we have recently reported that the *BDNF* Val66Met polymorphism modulates the FC dynamics in the DPMS and might substantiate individual vulnerability to the development of chronic functional pain in women with PDM^20^. Whether there is a gene–gene interaction between the *OPRM1* A118G polymorphism and the *BDNF* Val66Met polymorphism can be of high interest in further studies.

Multi-factorial models (including gene, sex, age, ethnicity, life event, psychology, and etc.) may underlie individual differences that make effective standardized-treatment difficult; however, studies have reported personalized opioid use by prediction formulas containing several genetic polymorphisms within or close to the *OPRM1* gene^45^. Our data also pinpoint the importance of individualizing analgesic therapy to optimize medical treatment for pain relief^46^. Given that the PAG is substantially involved in both placebo effects and motor cortex stimulation for pain alleviation^6^,^47^, AA homozygotes with higher functional expression of the DPMS may have better endogenous and exogenous modulations of pain. Our data suggest that conventional analgesic treatment or noninvasive brain stimulation might be good choices for chronic pain patients who are AA homozygotes. In contrast, analgesics combined with more aggressive “multimodal” pain alleviation techniques might be ideal choices for the chronic pain patients who are G allele carriers^46^.

## Conclusions

To our knowledge, this is the first study that provides novel insight into the hitherto unexplored neurodynamic influences of the *OPRM1* A118G polymorphism on the FCs of PAG-based DPMS. Such genetic variations shape the functional organization of DPMS and may predict or underpin the differential efficacy of analgesics (responder or non-responder)^48^, and eventually may also attribute to the vulnerability to the development of chronic pain late in life of PDM subjects^20^. Our preliminary report invites future studies of larger sample size for the verification and to further explore whether such *OPRM1* A118G polymorphism-FCs of PAG-seeded DPMS interactions for individual pain experience can be generalized to other chronic functional pain disorders.

## Materials and Methods

### Subjects

All participants were recruited from Internet advertisements from August 2011 to August 2015. Originally, 153 PDM subjects and 151 controls were screened using telephone and in-person structured interviews regardless of case or control status. Two PDM subjects and 7 controls were then excluded after rigorous screening according to the exclusion criteria and one of the following factors: inconsistent pain intensity, left-handedness, premenstrual dysphoric disorder, or head injury. After entering the integrated behavioral and multimodal imaging genetics studies (magnetic resonance imaging and magnetoencephalography), 8 PDM subjects were excluded owing to pelvic abnormalities on ultrasonography scans; 2 PDM subjects and 2 controls were excluded owing to an irregular menstrual cycle; 1 PDM subject and 1 control were excluded owing to a prolonged menstrual cycle; 17 PDM subjects and 2 controls were excluded owing to incidental brain findings^15^; 57 PDM subjects and 53 controls were excluded because they were unwilling to complete the entire series of genetic, hormonal, behavioral, and multimodal neuroimaging studies, which would resulted in incomplete datasets; 2 PDM subjects and 12 controls were excluded because of abnormal hormone levels (possibly due to sampling and technical errors); and 3 PDM subjects and 9 controls were excluded owing to head motion (>2 mm) or rotation (>2°) during the scan. Eventually, 61 PDM subjects and 65 controls were eligible for neuroimaging analyses in this study (entire protocol completed).

The inclusion criteria for the PDM subjects were the following: 1) a regular menstrual cycle of approximately 27–32 days; 2) a history of menstrual pain longer than 6 months; 3) an averaged menstrual pain under regular treatment with a rating that was higher than 4 on a verbal numerical scale (VNS, 0 = not at all, 10 = the worst imaginable pain) over the last 6 months; and 4) right-handedness, as confirmed by the Edinburgh Handedness Inventory. The inclusion criteria for the healthy female controls were similar to those for the PDM subjects, except that the controls had no pain whatsoever during menses (VNS = 0). The exclusion criteria for all the participants were as follows: 1) using oral contraceptives, hormonal supplements, Chinese medicine, or any centrally acting medication (e.g., opioid, anti-epileptics) within 6 months prior to the study; 2) pathological pituitary gland disease; 3) organic pelvic disease; 4) any psychiatric or neurological disorders; 5) any head injury with loss of consciousness; 6) immediate plans for pregnancy or a positive pregnancy test; 7) a history of childbirth; and 8) having a metal implant, a pacemaker implant, claustrophobia, or any contraindications in relation to magnetic resonance imaging (MRI). No analgesics had been taken by the subjects within 24 hours before the study. All PDM subjects were double-screened and diagnosed at a gynecology clinic by a gynecologist (H.T.C.) and received pelvic ultrasonography to exclude secondary dysmenorrhea caused by an organic pelvic disease such as endometriosis or adenomyosis. The study was conducted in accordance with the Declaration of Helsinki and was approved by the Institutional Review Board of Taipei Veterans General Hospital. All participants provided their written informed consent.

### Experimental design

Blood samples for genotyping were collected during the initial examination, but genotypes of all participants were not known before the scanning session. MRI scans (T1 and resting-state fMRI images) were individually scheduled according to each subject’s first day of menstruation at two time points during the menstrual cycle: menstruation (days 1–3 of the menstrual cycle) and peri-ovulatory phase (days 12–16 of the menstrual cycle). Blood samples were collected at the same day and subjected to a gonadal hormone assay. For the detailed information of the genotyping and serum gonadal hormone measurements, please refer to our published paper^7^,^20^.

PDM subjects completed the McGill Pain Questionnaire during the initial examination (for mnemonic pain) and menstruation (for present pain); all participants completed the thermal quantitative sensory tests (for experimental pain) during menstruation and peri-ovulatory phase. For mnemonic and present pain, the PRI value in the McGill Pain Questionnaire was used to access menstrual pain experience since the scoring encompasses different dimensions of pain experience (i.e., sensory, affective, evaluative, and miscellaneous dimensions). For experimental pain, heat-pain threshold (as an index of pain sensitivity) was assessed by means of thermal quantitative sensory test.

### Thermal quantitative sensory test

We performed thermal quantitative sensory testing according to the established protocol of an ascending limit approach for heat-pain thresholds^49^. Hot stimuli (TSA 2001-II, MEDOC, Israel) were administered to the bilateral peri-umbilical areas (T11-dermatome, referral area of menstrual pain) and forearm extensor areas (C7-dermatome, remote control area). The baseline temperature was set at 32 °C, and all the thresholds were obtained by a ramped stimulation method (1 °C/sec). The mean thresholds were calculated by averaging 3 consecutive measurements. The upper limit of the temperature was set at 50 °C.

### Image acquisition

Resting-state fMRI images were acquired using a 3.0 Tesla MRI scanner (Magnetom Trio Tim, Siemens, Erlangen, Germany) with a 12-channel head coil at National Yang-Ming University. High-resolution T1-weighted 3-dimensional structural images using a magnetization-prepared rapid-acquired gradient echo sequence (MPRAGE; TR/TE = 2530 ms/3.03 ms, flip angle = 70°, field-of-view = 224 × 256 × 192 mm^3^, matrix size = 224 × 256 × 192, in-plane resolution = 1 mm) and T2*-weighted gradient echo sequence (TR/TE = 2500 ms/30 ms, flip angle = 90°, field-of-view = 220 × 220 × 136 mm^3^, matrix size = 64 × 64 × 40, in-plane resolution = 3.4 mm [round-out], and 200 volumes per run) were performed to obtain high-resolution anatomical T1 images and fMRI images^7^. The participants remained awake during the scan (eyes open, head still but relaxed, without thinking about anything in particular). Head cushions and earplugs were provided to reduce head motion and noise, respectively.

### Image preprocessing

Preprocessing was performed using the DPARSF toolbox (State Key Laboratory of Cognitive Neuroscience and Learning, Beijing Normal University, China) with Statistical Parametrical Mapping 8 (SPM8, Wellcome Trust Centre for Neuroimaging, London, http://www.fil.ion.ucl.ac.uk/spm) in Matlab. All functional images were subjected to slice timing, realignment for head-motion correction, co-registration against each individual’s anatomical image as well as normalization (nonlinear transformation) against the Montreal Neurological Institute (MNI-152) template. Subjects exhibiting any head motion of more than 2 mm or 2° were excluded from further processing^50^. The images were re-sampled to an isotropic 2 × 2 × 2 mm^3^ voxel size during the normalization step and then spatially smoothed using a 3D Gaussian kernel of 8 mm full-width at half-maximum. Linear trends were then removed from the resulting time series, and the time series was temporally band-pass filtered (0.01–0.08 Hz) to extract the low-frequency oscillations associated with spontaneous neuronal activity^51^.

### Removal of physiological and scanner-related noise

The averaged time courses of the following nuisance variables or confounding artifacts were regressed out: 1) the six head-movement parameters computed based on rigid body translation and rotation during the realignment in SPM8; 2) the global mean signal (global signal regression); 3) the mean signal within the lateral ventricles; and 4) the mean signal within a deep white matter region (centrum ovale). The cerebrospinal fluid and the white-matter signals are thought to reflect fluctuations in non-specific regional correlations. We performed global signal regression because it can maximize the spatial specificity of positive resting-state correlations^52^, improve correspondence to anatomy^53^ and to electrophysiology^54^. The neuroscientific interpretation of anti-correlation has been challenged^55^, and global signal regression may cause a negative shift in the distribution of correlations^53^; therefore, we implemented a mask and addressed positive connectivity only in order to remove distortion after global signal regression^56^ as reported in our previously published work^7^,^20^.

### Definition of PAG seed and PAG-seeded FC maps

The PAG seed (3-mm radius), centered at MNI coordinate [−4, −26, −14], was identified based on the published literature^57^,^58^. This seed is located within the ventrolateral PAG^57^ that is important for the opioid-mediated analgesia^59^. In healthy subjects, this region was activated during increased levels of heat-pain stimulation^60^, and was functionally connected to the key regions of the DPMS^57^. The mean time-series activity in the seed region of each subject was extracted. PAG-seeded FC maps were then generated. Each individual-level FC map obtained was then converted into a z-map using Fisher’s *r*-to-*z* transformation for second-level group analyses^50^.

### Statistical analyses

#### Demographic information and psychophysiological measurements

SPSS Statistics 20.0 (SPSS Inc., Chicago, IL) was used for all of these analyses. The results were considered significant at *p* = 0.05 (two-tailed). The Hardy-Weinberg equilibrium of the *OPRM1* genotype distribution in each group and the A118G-genotype distribution between two groups were assessed using the chi-square test. Normality was tested for all assessments, and only the PRI values in the McGill Pain Questionnaire were normality (*p* > 0.05). Therefore, a repeated-measures analysis of variance (ANOVA) of the McGill Pain Questionnaire scores was conducted to assess the main effects of *OPRM1* genotype (AA homozygotes *vs*. G allele carriers) and pain-type (present *vs.* mnemonic), as well as the interaction between them. To assess the effects of groups (PDM *vs*. control) and/or *OPRM1* genotype (AA homozygotes *vs*. G allele carriers), a Mann–Whitney U test was conducted for demographic characteristics, Edinburgh Handedness Inventory scores, durations of menstrual pain, serum hormone levels, and heat-pain thresholds. To assess the effect of menstrual cycle phase (menstruation *vs.* peri-ovulatory phase), a Wilcoxon signed-rank test was applied to serum hormone levels and heat-pain thresholds. Generally, the data met the assumptions of the statistical tests used, and the variance was similar between groups and *OPRM1* genotypes.

#### Image analysis

A mixed-effects model of factorial design (2 factors: genotype and group) was employed to analyze the FC maps using SPM 8 (Wellcome Trust Centre for Neuroimaging, London, http://www.fil.ion.ucl.ac.uk/spm) for each phase. Gonadal hormones were regressed out as covariates of non-interest. Statistical maps (Fig. 1) were computed to identify changes in PAG-seeded FC for the following comparisons: 1) overall between-genotype comparison, 2) between-genotype comparisons for each group and 3) between-group comparisons for each genotype. PAG-seeded FC changes during menstruation (painful stage) in the comparisons were regarded as *state* changes, whereas changes during the peri-ovulatory phase (pain-free stage) or throughout the menstrual cycle were regarded as *trait* changes. Significance was thresholded at the uncorrected voxel level *p* = 0.005, followed by the FWE-corrected cluster level *p* = 0.05.

In the PDM group, the *state* correlation between the PRI of present pain and the PAG-seeded FC maps during menstruation was performed using a one-tailed two-sample *t*-test by the SPM. In contrast, the *trait* correlation between the PRI of mnemonic pain was similarly correlated with the PAG-seeded FC maps during the peri-ovulatory phase. We first entered the demeaned (in SPM) PRI values from present or mnemonic McGill Pain Questionnaire as regressors to identify brain regions with either positive or negative correlations with PAG-seeded FC in menstruation or peri-ovulatory phase^7^. Between-genotype comparisons were performed to identify regions where the correlation between PAG-seeded FC and the PRI was greater. Gonadal hormones were regressed out as covariates of non-interest. Significance was thresholded at the uncorrected voxel level *p* = 0.005, followed by the FWE-corrected cluster level *p* = 0.05.

For each phase, a one-tailed two-sample *t*-test was conducted to examine the correlation between the heat-pain thresholds and the PAG-seeded FC in the PDM group. Gonadal hormones were regressed out as covariates of non-interest. We first entered the demeaned (in SPM) heat-pain thresholds from the quantitative sensory test as regressors to identify brain regions with either positive or negative correlations with PAG-seeded FC in both genotypes. Between-genotype comparisons were performed to identify regions where the correlation between PAG-seeded FC and the heat-pain threshold was greater. Correlations during menstruation were regarded as *state* relationships, whereas correlations during the peri-ovulatory phase or throughout the menstrual cycle were regarded as *trait* relationships. Significance was thresholded at the uncorrected voxel level *p* = 0.005, followed by the FWE-corrected cluster level *p* = 0.05.

Because the SPM reports peak coordinates as identified within a confluent cluster, there can be multiple peaks that sit on different brain regions/areas (e.g., Brodmann area). We reported one representative peak for each region/area (e.g., in Table 4, dorsolateral prefrontal cortex and medial prefrontal cortex belong to a same cluster). Anatomical locations were determined according to probabilistic atlases of Harvard-Oxford Cortical Structural Atlas and Talairach Daemon Labels. To display 3D imaging, we used MRIcroGL for 3D rendering (Department of Psychology, University of South Carolina http://www.mccauslandcenter.sc.edu/mricrogl by Chris Rorden).

## Additional Information

**How to cite this article**: Wei, S.-Y. *et al*. The *OPRM1* A118G polymorphism modulates the descending pain modulatory system for individual pain experience in young women with primary dysmenorrhea. *Sci. Rep.* **7**, 39906; doi: 10.1038/srep39906 (2017).

**Publisher's note:** Springer Nature remains neutral with regard to jurisdictional claims in published maps and institutional affiliations.

## Figures and Tables

**Table 1:** Demographic data and baseline information.

	PDM (*n* = 61)	Control (*n* = 65)	*p* Value			
			Group	Genotype	Pain	Interaction
**Age, year**						
AA homozygotes	22.4 (2.61)	23.2 (1.64)	0.087	0.408	NA	NA
G allele carriers	23.0 (2.04)	24.2 (2.68)				
**Years of menstruating**						
AA homozygotes	11.3 (2.69)	10.5 (1.49)	0.484	0.325	NA	NA
G allele carriers	10.9 (2.60)	12.0 (3.16)				
**Days of one menstrual cycle**						
AA homozygotes	29.4 (1.55)	29.5 (1.25)	0.546	0.760	NA	NA
G allele carriers	29.5 (1.48)	29.5 (1.53)				
**Edinburgh Handedness**						
AA homozygotes	82.5 (17.63)	85.6 (14.35)	0.643	0.291	NA	NA
G allele carriers	82.0 (17.81)	77.3 (20.53)				
Mnemonic PRI scores*						
AA homozygotes	34.7 (12.75)	NA	NA	0.531	0.039	0.300
G allele carriers	35.0 (14.88)	NA				
Present PRI scores*						
AA homozygotes	29.5 (12.94)	NA				
G allele carriers	33.2 (13.04)	NA				

Normality was tested for all assessments, and only the PRI scores in the McGill Pain Questionnaire were normality (*p* > 0.05). The data are presented as the means (SD). NA, not applicable.

^*^Four subjects with primary dysmenorrhea (PDM; 3 AA homozygotes, 1 G allele carrier) did not complete the pain rating index (PRI) of the McGill Pain Questionnaire and were excluded from these calculations.

**Table 2:** Results of gonadal hormone levels: effects of group, genotype and menstrual cycle phase.

	PDM		Control		*p* Value		
	AA	G carrier	AA	G carrier	Phase	Group	Genotype
	(*n* = 26)	(*n* = 35)	(*n* = 23)	(*n* = 42)			
**Estradiol (pg/mL)**							
Menstruation	32.9 (14.12)	33.4 (17.89)	30.2 (15.53)	37.2 (17.96)	0.000	0.653	0.166
Peri-ovulatory phase	146.9 (84.23)	157.5 (115.81)	151.8 (132.23)	142.8 (114.04)		0.370	0.770
**Progesterone (ng/mL)**							
Menstruation	0.5 (0.46)	0.4 (0.25)	0.9 (2.50)	0.5 (0.30)	0.007	0.396	0.857
Peri-ovulatory phase	0.9 (1.38)	0.7 (0.79)	1.9 (3.23)	1.3 (2.78)		0.709	0.625
**Testosterone (ng/mL)**							
Menstruation	0.4 (0.28)	0.3 (0.17)	0.4 (0.22)	0.4 (0.22)	0.000	0.830	0.743
Peri-ovulatory phase	0.5 (0.37)	0.5 (0.23)	0.4 (0.24)	0.5 (0.18)		0.862	0.707

The data are presented as the means (SD). PDM, primary dysmenorrhea.

**Table 3:** Results of heat-pain threshold: effects of group, genotype and menstrual cycle phase.

	PDM		Control		*p* Value		
	AA	G carrier	AA	G carrier	Phase	Group	Genotype
**C7-dermatome**							
Menstruation	43.7 (3.65)	44.4 (3.07)	46.0 (2.74)	45.0 (3.69)	0.231	0.024	0.938
Peri-ovulatory phase	43.3 (3.04)	44.3 (2.95)	46.3 (2.20)	44.6 (3.59)		0.019	0.865
**T11-dermatome**							
Menstruation	42.8 (3.12)	43.4 (2.75)	44.7 (2.96)	43.8 (3.49)	0.599	0.130	0.831
Peri-ovulatory phase	42.8 (2.99)	43.6 (2.69)	45.1 (2.83)	43.8 (3.66)		0.094	0.883

Subjects who did not complete the thermal quantitative sensory test were excluded from respective calculations: 1 PDM subject with G allele during menstruation, 2 PDM subjects with AA homozygotes during the peri-ovulatory phase, 1 control with G allele during menstruation, and 3 controls with AA homozygotes during menstruation.

The data are presented as the means (SD). PDM, primary dysmenorrhea.

**Table 4:** Peak MNI coordinates of the regions exhibiting significant resting-state FC with the PAG for the between-genotype and between-group differences.

Contrast	Phase	Region	BA	Cluster	*t* Score	Peak coordinate		
						*x*	*y*	*z*
G allele carriers overall < AA homozygotes overall	Menstruation	mPFC	9	1227	3.92	14	44	32
		dlPFC	8	—	3.02	48	26	44
		ACC	32	968	3.67	−14	36	18
G allele carrier controls < AA homozygous controls	Menstruation	dlPFC	8	452	3.84	38	36	46
		Temporal	21	588	3.58	60	−36	−12
		ACC	32	387	3.19	−18	42	10
G allele carrier PDMs < AA homozygous PDMs	Menstruation	Occipital	19	151	4.04	−38	−80	14
		SPL	7	104	3.53	26	−76	54
		Orbitofrontal	11	63	3.36	14	30	−22
		ACC	24	165	3.11	14	−4	42
		mPFC	9	60	2.95	18	40	32
G allele carrier PDMs < AA homozygous PDMs	Peri-ovulatory phase	Cerebellum	—	443	4.02	−26	−54	−56
G allele carrier PDMs > G allele carrier controls	Menstruation	SMA	6	566	4.51	2	−18	56

Peak coordinates refer to the Montreal Neurological Institute space. Significance was thresholded at the uncorrected voxel level *p* = 0.005, followed by the FWE-corrected cluster level *p* = 0.05, except the between-genotype comparison for PDM subjects. The contrast of G allele carrier PDMs < AA homozygous PDMs was thresholded at the uncorrected voxel level *p* = 0.005, voxels > 60. ACC, anterior cingulate cortex; BA, Brodmann area; dlPFC, dorsolateral prefrontal cortex; mPFC, medial prefrontal cortex; SMA, supplementary motor area; SPL, superior parietal lobule.

**Table 5:** PAG functional connectivity co-varying with the present pain rating index of the primary dysmenorrheic subjects.

Phase	Genotype	Region	BA	Cluster	*t* Score	Peak coordinate		
						*x*	*y*	*z*
Menstruation	G allele carriers	NA	NA	NS	NS	NA	NA	NA
		**Positive**						
Menstruation	AA homozygotes	Insula	13	11113	4.58	38	−10	4
		Anterior cingulate cortex	24	—	4.44	−10	4	38
		Anterior cingulate cortex	32	—	4.40	−10	22	40
		Secondary somatosensory	44	—	4.26	52	10	0
		Subthalamic nucleus	—	—	4.05	12	−16	−8
		Cerebellum	—	—	4.04	−12	−60	−30

Peak coordinates refer to the Montreal Neurological Institute (MNI) space. Significance was thresholded at the uncorrected voxel level *p* = 0.005, followed by the FWE-corrected cluster level *p* = 0.05. BA, Brodmann area; mPFC, medial prefrontal cortex; NA, not available; NS, not significant.

^*^Four subjects with primary dysmenorrhea (3 AA homozygotes, 1 G allele carrier) did not complete the McGill Pain Questionnaire for present menstrual pain and were excluded from this calculation.

**Table 6:** PAG functional connectivity co-varying with the heat-pain threshold of the primary dysmenorrheic subjects.

Phase	Dermatome	Genotype	Region	BA	Cluster	*t* Score	Peak coordinate		
							*x*	*y*	*z*
Menstruation*			**Negative**						
	T11	G allele carriers	Cuneus	19	637	5.03	8	−88	30
			**Negative**						
	C7	G allele carriers	Cuneus	18	639	3.61	−2	−88	28
Peri-ovulatory phase**			**Negative**						
	T11	G allele carriers	Cuneus	17	2038	3.67	8	−86	6
			**Negative**						
	C7	G allele carriers	Cuneus	18	1303	3.84	−8	−96	20

Peak coordinates refer to the Montreal Neurological Institute (MNI) space. Significance was thresholded at the uncorrected voxel level *p* = 0.005, followed by the FWE-corrected cluster level *p* = 0.05. BA, Brodmann area.

^*^One subject with primary dysmenorrhea did not finish the quantitative sensory test and was excluded from this calculation.

^**^Two subjects with primary dysmenorrhea did not finish the quantitative sensory test and were excluded from this calculation.

## References

[b1] Fields H. State-dependent opioid control of pain. Nat Rev Neurosci 5, 565–575 (2004). 15208698 10.1038/nrn1431

[b2] Zhang Y., Wang D., Johnson A. D., Papp A. C. & Sadee W. Allelic expression imbalance of human mu opioid receptor (OPRM1) caused by variant A118G. J Biol Chem 280, 32618–32624 (2005). 16046395 10.1074/jbc.M504942200

[b3] Yao P. . Effect of gene polymorphism of COMT and OPRM1 on the preoperative pain sensitivity in patients with cancer. Int J Clin Exp Med 8, 10036–10039 (2015). 26309696 PMC4538055

[b4] Sia A. T. . A118G single nucleotide polymorphism of human mu-opioid receptor gene influences pain perception and patient-controlled intravenous morphine consumption after intrathecal morphine for postcesarean analgesia. Anesthesiology 109, 520–526 (2008). 18719451 10.1097/ALN.0b013e318182af21

[b5] Carrive P. & Morgan M. M. Periaqueductal Gray. In The Human Nervous System (ed. Mai J. K. & Paxinos G.) 367–400 (Academic Press, Waltham (MA), 2011).

[b6] Wager T. D., Scott D. J. & Zubieta J. K. Placebo effects on human mu-opioid activity during pain. Proc Natl Acad Sci USA 104, 11056–11061 (2007). 17578917 10.1073/pnas.0702413104 PMC1894566

[b7] Wei S. Y. . Changes in functional connectivity of pain modulatory systems in women with primary dysmenorrhea. Pain 157, 92–102 (2016). 26307856 10.1097/j.pain.0000000000000340

[b8] Berkley K. J. Primary Dysmenorrhea: An Urgent Mandate. Pain: Clinical Updates 21, 1–8 (2013).

[b9] Tu C. H. . Abnormal cerebral metabolism during menstrual pain in primary dysmenorrhea. Neuroimage 47, 28–35 (2009). 19362153 10.1016/j.neuroimage.2009.03.080

[b10] Tu C. H. . Brain morphological changes associated with cyclic menstrual pain. Pain 150, 462–468 (2010). 20705214 10.1016/j.pain.2010.05.026

[b11] Tu C. H. . Menstrual pain is associated with rapid structural alterations in the brain. Pain 154, 1718–1724 (2013). 23693160 10.1016/j.pain.2013.05.022

[b12] Wu T. H. . Dynamic Changes of Functional Pain Connectome in Women with Primary Dysmenorrhea. Sci Rep 6, 24543 (2016). 27089970 10.1038/srep24543 PMC4835697

[b13] Liu P. . Aberrant default mode network in patients with primary dysmenorrhea: a fMRI study. Brain Imaging Behav (2016). 10.1007/s11682-016-9627-1 27738992

[b14] Kutch J. J. & Tu F. F. Altered brain connectivity in dysmenorrhea: pain modulation and the motor cortex. Pain 157, 5–6 (2016). 26683107 10.1097/j.pain.0000000000000364 PMC4941100

[b15] Li W. C. . High prevalence of incidental brain findings in primary dysmenorrhoea. Eur J Pain 19, 1071–1074 (2015). 25487523 10.1002/ejp.639

[b16] Yu R. . Disrupted functional connectivity of the periaqueductal gray in chronic low back pain. Neuroimage Clin 6, 100–108 (2014). 25379421 10.1016/j.nicl.2014.08.019 PMC4215524

[b17] Michels L. . Pain modulation is affected differently in medication-overuse headache and chronic myofascial pain - A multimodal MRI study. Cephalalgia (2016). 10.1177/0333102416652625 27250235

[b18] Truini A. . Abnormal resting state functional connectivity of the periaqueductal grey in patients with fibromyalgia. Clin Exp Rheumatol 34, S129–133 (2016). 27157397

[b19] Li Z. . Altered periaqueductal gray resting state functional connectivity in migraine and the modulation effect of treatment. Sci Rep 6, 20298 (2016). 26839078 10.1038/srep20298 PMC4738255

[b20] Wei S. Y. . The BDNF Val66Met polymorphism is associated with the functional connectivity dynamics of pain modulatory systems in primary dysmenorrhea. Sci. Rep. 6, 23639 (2016). 27010666 10.1038/srep23639 PMC4806293

[b21] Everts B. . Pain recollection after chest pain of cardiac origin. Cardiology 92, 115–120 (1999). 10702654 10.1159/000006958

[b22] Vincent K. . Brain imaging reveals that engagement of descending inhibitory pain pathways in healthy women in a low endogenous estradiol state varies with testosterone. Pain 154, 515–524 (2013). 23318125 10.1016/j.pain.2012.11.016

[b23] Bartley E. J. . Natural variation in testosterone is associated with hypoalgesia in healthy women. Clin J Pain 31, 730–739 (2015). 25185874 10.1097/AJP.0000000000000153

[b24] Petersen N., Kilpatrick L. A., Goharzad A. & Cahill L. Oral contraceptive pill use and menstrual cycle phase are associated with altered resting state functional connectivity. Neuroimage 90, 24–32 (2014). 24365676 10.1016/j.neuroimage.2013.12.016 PMC4113343

[b25] Giamberardino M. A., Berkley K. J., Iezzi S., de Bigontina P. & Vecchiet L. Pain threshold variations in somatic wall tissues as a function of menstrual cycle, segmental site and tissue depth in non-dysmenorrheic women, dysmenorrheic women and men. Pain 71, 187–197 (1997). 9211480 10.1016/s0304-3959(97)03362-9

[b26] Bajaj P., Bajaj P., Madsen H. & Arendt-Nielsen L. A comparison of modality-specific somatosensory changes during menstruation in dysmenorrheic and nondysmenorrheic women. Clin J Pain 18, 180–190 (2002). 12048420 10.1097/00002508-200205000-00007

[b27] Brinkert W., Dimcevski G., Arendt-Nielsen L., Drewes A. M. & Wilder-Smith O. H. Dysmenorrhoea is associated with hypersensitivity in the sigmoid colon and rectum. Pain 132 Suppl 1, S46–51 (2007). 17257758 10.1016/j.pain.2006.12.011

[b28] Garcia-Larrea L. & Peyron R. Pain matrices and neuropathic pain matrices: a review. Pain 154 Suppl 1, S29–43 (2013). 24021862 10.1016/j.pain.2013.09.001

[b29] Ren Z. Y. . The impact of genetic variation on sensitivity to opioid analgesics in patients with postoperative pain: a systematic review and meta-analysis. Pain Physician 18, 131–152 (2015). 25794200

[b30] Vogt B. A., Derbyshire S. & Jones A. K. Pain processing in four regions of human cingulate cortex localized with co-registered PET and MR imaging. Eur J Neurosci 8, 1461–1473 (1996). 8758953 10.1111/j.1460-9568.1996.tb01608.x

[b31] Petrovic P., Kalso E., Petersson K. M. & Ingvar M. Placebo and opioid analgesia– imaging a shared neuronal network. Science 295, 1737–1740 (2002). 11834781 10.1126/science.1067176

[b32] Eippert F. . Activation of the opioidergic descending pain control system underlies placebo analgesia. Neuron 63, 533–543 (2009). 19709634 10.1016/j.neuron.2009.07.014

[b33] Kucyi A., Salomons T. V. & Davis K. D. Mind wandering away from pain dynamically engages antinociceptive and default mode brain networks. Proc Natl Acad Sci USA 110, 18692–18697 (2013). 24167282 10.1073/pnas.1312902110 PMC3832014

[b34] Valet M. . Distraction modulates connectivity of the cingulo-frontal cortex and the midbrain during pain–an fMRI analysis. Pain 109, 399–408 (2004). 15157701 10.1016/j.pain.2004.02.033

[b35] Bonenberger M., Plener P. L., Groschwitz R. C., Gron G. & Abler B. Polymorphism in the micro-opioid receptor gene (OPRM1) modulates neural processing of physical pain, social rejection and error processing. Exp Brain Res 233, 2517–2526 (2015). 26019010 10.1007/s00221-015-4322-9

[b36] Reis G. M. . Antinociceptive effect of stimulating the occipital or retrosplenial cortex in rats. J Pain 11, 1015–1026 (2010). 20418174 10.1016/j.jpain.2010.01.269

[b37] Fillingim R. B. . The A118G single nucleotide polymorphism of the mu-opioid receptor gene (OPRM1) is associated with pressure pain sensitivity in humans. J Pain 6, 159–167 (2005). 15772909 10.1016/j.jpain.2004.11.008

[b38] Lopez Soto E. J. & Catanesi C. I. Human population genetic structure detected by pain-related mu opioid receptor gene polymorphisms. Genet Mol Biol 38, 152–155 (2015). 26273217 10.1590/S1415-4757382220140299 PMC4530646

[b39] Chen D. . Ethnic-specific meta-analyses of association between the OPRM1 A118G polymorphism and alcohol dependence among Asians and Caucasians. Drug Alcohol Depend 123, 1–6 (2012). 22071118 10.1016/j.drugalcdep.2011.10.012

[b40] Rasetti R. & Weinberger D. R. Intermediate phenotypes in psychiatric disorders. Curr Opin Genet Dev 21, 340–348 (2011). 21376566 10.1016/j.gde.2011.02.003 PMC3138621

[b41] Denk F., McMahon S. B. & Tracey I. Pain vulnerability: a neurobiological perspective. Nat Neurosci 17, 192–200 (2014). 24473267 10.1038/nn.3628

[b42] Lui S. . High-field MRI reveals an acute impact on brain function in survivors of the magnitude 8.0 earthquake in China. Proc Natl Acad Sci USA 106, 15412–15417 (2009). 19720989 10.1073/pnas.0812751106 PMC2735557

[b43] Lister R. . Global epigenomic reconfiguration during mammalian brain development. Science 341, 1237905 (2013). 23828890 10.1126/science.1237905 PMC3785061

[b44] Hyman S. E. The genetics of mental illness: implications for practice. Bull World Health Organ 78, 455–463 (2000). 10885164 PMC2560734

[b45] Yoshida K. . Prediction formulas for individual opioid analgesic requirements based on genetic polymorphism analyses. PLoS One 10, e0116885 (2015). 25615449 10.1371/journal.pone.0116885 PMC4304713

[b46] Hajj A. . Genotyping test with clinical factors: better management of acute postoperative pain? Int J Mol Sci 16, 6298–6311 (2015). 25809606 10.3390/ijms16036298 PMC4394533

[b47] DosSantos M. F. . Building up analgesia in humans via the endogenous mu-opioid system by combining placebo and active tDCS: a preliminary report. PLoS One 9, e102350 (2014). 25029273 10.1371/journal.pone.0102350 PMC4100885

[b48] Trescot A. M. & Faynboym S. A review of the role of genetic testing in pain medicine. Pain Physician 17, 425–445 (2014). 25247900

[b49] Rolke R. . Quantitative sensory testing: a comprehensive protocol for clinical trials. Eur J Pain 10, 77–88 (2006). 16291301 10.1016/j.ejpain.2005.02.003

[b50] Chao-Gan Y. & Yu-Feng Z. DPARSF: A MATLAB Toolbox for “Pipeline” Data Analysis of Resting-State fMRI. Front Syst Neurosci 4, 13 (2010). 20577591 10.3389/fnsys.2010.00013 PMC2889691

[b51] Lu H. . Synchronized delta oscillations correlate with the resting-state functional MRI signal. Proc Natl Acad Sci USA 104, 18265–18269 (2007). 17991778 10.1073/pnas.0705791104 PMC2084331

[b52] Weissenbacher A. . Correlations and anticorrelations in resting-state functional connectivity MRI: a quantitative comparison of preprocessing strategies. Neuroimage 47, 1408–1416 (2009). 19442749 10.1016/j.neuroimage.2009.05.005

[b53] Fox M. D., Zhang D., Snyder A. Z. & Raichle M. E. The global signal and observed anticorrelated resting state brain networks. J Neurophysiol 101, 3270–3283 (2009). 19339462 10.1152/jn.90777.2008 PMC2694109

[b54] Keller C. J. . Neurophysiological investigation of spontaneous correlated and anticorrelated fluctuations of the BOLD signal. J Neurosci 33, 6333–6342 (2013). 23575832 10.1523/JNEUROSCI.4837-12.2013 PMC3652257

[b55] Buckner R. L., Krienen F. M. & Yeo B. T. Opportunities and limitations of intrinsic functional connectivity MRI. Nat Neurosci 16, 832–837 (2013). 23799476 10.1038/nn.3423

[b56] Chen Y. L. . Resting-state fMRI mapping of cerebellar functional dysconnections involving multiple large-scale networks in patients with schizophrenia. Schizophr Res 149, 26–34 (2013). 23810119 10.1016/j.schres.2013.05.029

[b57] Kong J., Tu P. C., Zyloney C. & Su T. P. Intrinsic functional connectivity of the periaqueductal gray, a resting fMRI study. Behav Brain Res 211, 215–219 (2010). 20347878 10.1016/j.bbr.2010.03.042 PMC2862838

[b58] Linnman C., Beucke J. C., Jensen K. B., Gollub R. L. & Kong J. Sex similarities and differences in pain-related periaqueductal gray connectivity. Pain 153, 444–454 (2012). 22154332 10.1016/j.pain.2011.11.006 PMC3558685

[b59] Behbehani M. M. Functional characteristics of the midbrain periaqueductal gray. Prog Neurobiol 46, 575–605 (1995). 8545545 10.1016/0301-0082(95)00009-k

[b60] Kong J. . Exploring the brain in pain: activations, deactivations and their relation. Pain 148, 257–267 (2010). 20005043 10.1016/j.pain.2009.11.008 PMC2815185

